# The Immunological Role of Vascular and Lymphatic Endothelial Cells in Filarial Infections

**DOI:** 10.3390/ani12040426

**Published:** 2022-02-10

**Authors:** Magdalena Elżbieta Wysmołek, Ewa Długosz, Marcin Wiśniewski

**Affiliations:** Division of Parasitology and Parasitic Diseases, Department of Preclinical Sciences, Institute of Veterinary Medicine, Warsaw University of Life Sciences—SGGW, 02-786 Warsaw, Poland; ewa_dlugosz@sggw.edu.pl (E.D.); marcin_wisniewski@sggw.edu.pl (M.W.)

**Keywords:** immune response, epithelial cells, endothelium, cross-talk filarial nematodes–endothelium, host–parasite interactions, *Brugia malayi*, *Dirofilaria immitis*, VEGF, plasmin, ICAM, VCAM, PECAM, CD31

## Abstract

**Simple Summary:**

The endothelium is a monolayer of cells forming a thin membrane that lines the inside of blood vessels. These cells release molecules that regulate vascular relaxation, contraction, and can control blood clotting and the immune response. During infections with filarial nematodes, common parasites of humans and animals, the endothelium is believed to play a key role in the communication between the host and the parasite, since the embryonic stage of filaroids is distributed in the bloodstream. Therefore, this review aims to gather research from different scientists in order to better understand the host immune response in infections with filarial nematodes.

**Abstract:**

The embryonic stage of filarial nematodes, or microfilariae (Mf), shows daily and seasonal periodicity that requires their migration through blood vessels into the lungs, where they are sequestered when not circulating in the peripheral blood. Therefore, Mf and the host endothelium are likely in a permanent state of hide and seek. Interestingly, filarial nematodes co-cultured in media with a murine endothelial cell line survive eight times longer than those cultured in media alone. This suggests that the endothelium is an important element of the immune response in filarial nematodes, perversely promoting their survival in the host. In this review, we will focus on potential pathways involved in the relationship between filarial nematodes and the host endothelium, including the role of endothelial ICAM/VCAM/PECAM adhesion molecules, surface markers involved in the passage of Mf through host tissue, anti-thrombolic effects caused by the presence of filarial nematodes (including plasmins), endothelial cell proliferation (VEGF), and other aspects of the immune activation of the endothelium. The aim of this review is to merge the knowledge about the cross-talk between Mf of different filarial nematode species and endothelial cells (EC), thus allowing a better understanding of the mechanism of these parasitic infections.

## 1. Introduction

Parasitic filarial nematode infections are considered a rare disease; however, according to the World Health Organization (WHO), in 2017 alone there were 14.6 million people infected with onchocerciasis presented with skin disease and 1.15 million with vision loss [1]. Infection by *Dirofilaria repens* and *Dirofilaria immitis* is the leading cause of canine filariasis in Asia, Europe, and North America. These parasites are zoonotic, and an increasing number of infections of humans has been reported [2,3,4]. For example, 1200 human cases of *Dirofilaria* spp. infections were registered in the territory of the ex-USSR during the period of 1915–2016 [5], 40 cases were reported in Austria [6], and 1465 in Ukraine [7]. In the tropical regions of the world, lymphatic filariasis is primarily caused by *Brugia malayi* and *Wuchereria bancrofti*, and is a continuing problem as 859 million people in 50 countries worldwide remain at risk of infection, and 51 million people are estimated to be infected, according to the WHO [8]. Thus, extensive research on the development of vaccines against filarial nematodes is ongoing [9].

Filariasis includes vector-borne diseases caused by a group of roundworms called filarial nematodes. The pre-L1 larvae, called microfilariae (Mf), are taken up by blood-sucking insects during a blood meal. In a timescale of weeks, Mf molt in the arthropod until they reach the L3 stage, and are injected into the final host during a subsequent blood meal, where they reach maturity, copulate, and the females release Mf into the host bloodstream. There are three phenotypes of filariasis: lymphatic (*Wuchereria bancrofti*, *Brugia malayi*), subcutaneous (*Onchocerca volvulus*, *Diforilaria repens*), or serous cavity (*Dirofilaria immitis*). Briefly, lymphatic filariasis impairs the lymphatic system, which is characterized by lymphedema, elephantiasis, and, in men, swelling of the scrotum, called hydrocele; all of these lead to severe disability in humans. Onchocerciasis, also known as river blindness, is associated with dermatitis and can occur with a loss of sight in humans as a result of a strong inflammatory response against *O. volvulus* antigens migrating through the corneal endothelium. Finally, *D. immitis* mainly affects dogs and is a causative agent of heartworm disease, leading to heart failure in canines. Treatments in use can have efficacity against adult worms, such as diethylcarbamazine (DEC) [10,11], or show microfilaricidal activity, such as ivermectin [11,12,13]. Additionally, recent evidence suggests that tetracyclines have both microfilaricidal and macrofilaricidal effects against filaroids [14,15,16].

Many species of filarial nematode require the migration of Mf through blood vessels and into the lung capillaries during their daily or seasonal periodicities. This leads to permanent interactions between Mf and the host endothelial cells (EC). As a result, a complex relationship between host EC and the molecules of the invading filarial nematodes is established. Evans et al. observed that Mf co-cultured in media with a murine endothelial cell line survived eight times longer than those cultured in media alone. This suggests that the endothelium is an important element of the immune response to filaroids, perversely promoting their survival in the host [17]. The appearance of Mf in the bloodstream follows a circadian periodicity regulated by light. Mf reach their peak concentration in the peripheral blood during the hours when mosquitos are most likely to feed [18], which is supported by a finding from Hayasaki et al., who observed that *D. immitis* Mf show positive phototaxis toward infrared light [19].

The adhesion of leukocytes to vascular EC is a hallmark of the inflammation process and the host’s defense against pathogens [20]. On the other hand, in non-inflamed tissues during homeostasis, vascular EC maintain blood fluidity, regulate blood flow, control vessel wall permeability, and quiesce circulating leukocytes, as reviewed by Yang et al. [21,22]. In light of the crucial importance of EC in response to bloodborne parasites, an understanding of parasite–EC interactions may elucidate the mechanisms of innate immunity during filariasis.

In this review, we will focus on four potential pathways involved in the relationship between filarial nematodes and host EC, including the role of endothelial intercellular adhesion molecule 1 (ICAM)/vascular cell adhesion protein 1 (VCAM)/platelet endothelial cell adhesion molecule 1 (PECAM, CD31) adhesion molecules, surface markers involved in the passage of Mf through host tissue, anti-thrombolic effects caused by the presence of filarial nematodes (including plasmins), endothelial cell proliferation (VEGF), and other aspects of the immune activation of the endothelium. The aim of this review is to merge the knowledge concerning different species of filarial nematodes’ cross-talk with EC, thus allowing for a better understanding of the mechanism of these parasitic infections.

## 2. Migration/Adherence

ICAM-1, ICAM-2, VCAM-1, and CD31/PECAM-1 all belong to an immunoglobulin superfamily that is expressed on the surface of EC and plays a crucial role in migration and adherence in host–parasite cross-talk. CD31/PECAM-1 was firstly described by Muller et al. [23], and its role in trans-endothelial migration was more thoroughly evaluated a few years later [24,25,26]. ICAM [27] is not highly expressed in normal tissue, but inflammatory stimuli, such as tumor necrosis factor alfa (TNFα), interferon gamma (IFN-γ), and bacterial endotoxin, significantly upregulate its expression and cell surface localization [28]. Thus, the up-regulation of ICAM-1 production on the vascular endothelium permits leukocyte adhesion and represents an early stage in the migration of these cells into sites of tissue inflammation by extravasation [29,30]. Finally, the vascular cell adhesion molecule VCAM was first identified as a cytokine-inducible adhesion molecule on endothelium, mediating the interactions of EC with tumor cells and leukocytes [30,31,32]. These molecules interact with leukocyte counter-receptors to mediate the firm adhesion of leukocytes and their trans-endothelial migration in normally functioning endothelia during both homeostasis and inflammation [20]. Another important family of cell adhesion molecules is selectins, which bind sugar polymers, providing cell adhesion. As the leukocyte rolls along the blood vessel wall, the distal lectin-like domain of the selectin binds to certain carbohydrate groups presented on the proteins on the leukocyte, which slows the cell and allows it to leave the blood vessel to enter the site of infection. Selectins show a low-affinity binding that allows the characteristic “rolling” action attributed to leukocytes during the leukocyte adhesion cascade [33].

Importantly, adhesion to EC is not a property exclusive to host cells, but can also be presented by parasites, described sequentially in the next sections. Filarial nematodes, known for being masters of modulating the host immune response, likely use the upregulation of adhesion molecules expressed on the endothelium to freely move from the peripheral blood to the lungs’ capillaries, with respect to their circadian periodicity [18]. Filarial nematodes also likely use adhesion molecules to not only bind, but also to modulate the populations of leukocytes that can transmigrate through the endothelium.

### 2.1. Brugia malayi

**Figure 1:**
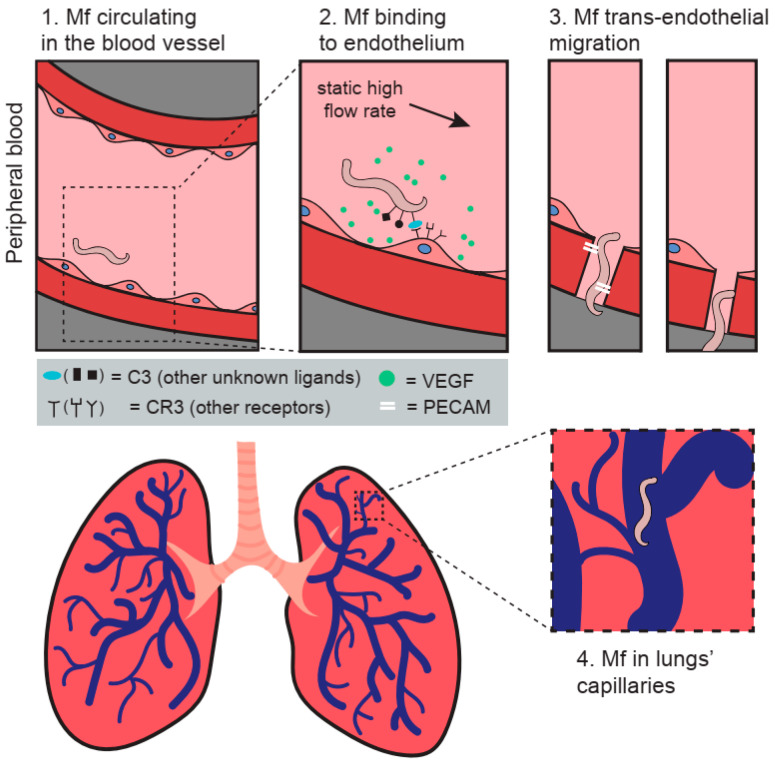
The figure presents a model of the potential trans-endothelial migration mechanisms adopted by Mf in the host. (**1**) Mf are circulating in the bloodstream during mosquito feeding time. (**2**) Increase in flow rate and the release of VEGF and likely other molecules promote the biding of Mf to the endothelium through complement component 3. (**3**) Mf trans-migration through the endothelium is likely enabled by CD31/PECAM-1. (**4**) Mf are sequestered in the lungs’ capillaries in respect to their circadian periodicity. The figure was designed and created by MEW and Paulina Wysmołek.

It has been reported that, in an athymic mouse infected with *B. malayi*, Mf adhered to EC with their anterior end [34] that was originally defined as a “hook” [35]. Since then, Schroeder et al. showed that live *B. malayi* Mf inhibit the in vitro trans-endothelial migration of neutrophils and monocytes, but not lymphocytes [36]. In the same paper, they also reported that, when the endothelium was exposed to Mf, the vascular EC expression of key mediators of the tethering stage of extravasation, such as ICAM-1 and VCAM-1, was unchanged. These studies concluded that the EC surface expression of VCAM-1, P-selectin, E-selectin, and ICAM-1 was not involved in Mf binding and the question around the mechanism of cross-talk between Mf and EC remained unresolved [36,37]. A few years later, Schroeder et al. observed that *B. malayi* Mf adhered to human umbilical vein endothelial cells (HUVEC) and TNF-α activated THP-1 cells, but not HEK 293 cells [38]. Further experiments involved HUVEC incubated with Mf in the presence of either complement C3-depleted human serum or untreated human serum. Schroeder et al. observed that the adherence of Mf to the endothelium was significantly decreased in C3-depleted serum compared to untreated serum, indicating that C3 plays a key role in Mf binding [38] (Figure 1). C3 depositions were detected on the surface of Mf co-cultured with intact serum, particularly concentrated at the anterior and posterior ends of Mf. Therefore, C3b and/or iC3b are suspected to bind to the microfilarial sheath in order to play a role as ligands for C3 receptors expressed on the endothelium. Furthermore, iC3b and Factor H (which can lead to iC3b formation) have been found on the surface of Mf from both *O. volvulus* and *Loa loa* after in vitro treatment with human serum [39,40]. Others have reported that *B. malayi* Mf secrete potent vasodilators, such as prostacyclin and prostaglandin E2 (PGE2), which may allow these parasites to modulate the vasculature function of the endothelium [41,42]. By showing that *B. malayi* Mf can bind to vascular EC in vitro, a hypothesis can be raised that the active adherence of Mf to EC may be a key mechanism by which Mf sequester in the lungs of the human host during times when mosquitos are not feeding [18,43]. As for periodicity, it has been reported that *B. malayi* Mf bind to the vascular EC under static conditions and under high, but not low, flow rates [38]. 

Schroeder et al. also tested different stimuli that are believed to influence the periodicity of parasites, but neither the addition of melatonin (at concentrations analogous to the ones during sleep in humans), nor treatment with the antiparasitic drug diethylcarbamazine (DEC), nor a drop in temperature had a direct effect upon microfilarial binding to HUVEC in vitro. Only when the cells were incubated in a hypoxic environment was the adherence of Mf to the EC surface significantly lower [38].

Weinkopff and Lammie [44] investigated the effect of adult and Mf *B. malayi* excretory–secretory products (ES) on the activation of human lymphatic EC (hLEC). However, after implementing a variety of approaches, including cellular proliferation, cell surface molecule expression, and cytokine and growth factor production, they did not observe any direct effect of the parasites’ proteins on the hLEC. Nevertheless, monocytes/macrophages exposed to filarial ES products secreted soluble VEGF that stimulated vessel growth associated with the pathogenesis of filarial disease [44].

### 2.2. Ligmosoides sigmodontis

Schroeder et al. [38] also tested if all Mf displayed the ability to adhere to EC or if it was reserved exclusively to periodic filarial nematodes. They observed that *L. sigmodontis* showed scarce adherence to HUVEC and no adherence to HEK 293T cell line.

### 2.3. Wuchereria bancrofti

Esterre et al. [45] compared the serum concentrations of soluble ICAM-1 and VCAM-1 in human patients suffering from lymphangitis of different origins—filarial or bacterial—but they did not observe any alterations specific only to lymphangitis related to *W. bancrofti* infection. However, in patients presenting elephantiasis or hydrocele, the levels of soluble L-selectin (leukocytes’ cell adhesion molecule) were decreased, while the levels of ET-1 (endothelial vasoconstrictor) were increased.

### 2.4. Onchocerca volvulus

Kaifi et al. [46] determined the expression of cell adhesion molecules in response to onchocerciasis using a murine model in which the antigens from *O. volvulus* were injected into the corneal stroma. They reported that the expression of each of these molecules was elevated after the injection of parasite antigens; CD31/PECAM-1 and ICAM-1 expression remained elevated from 12 h after injection until 7 days, whereas VCAM-1 expression was more transient, with peak expression at 72 h. After performing a VCAM-1 blockade, they did not observe any significant difference in corneal opacification. However, they observed that CD31/PECAM-1 played an important role in the recruitment of neutrophils. On the other hand, the ICAM-1 blockade resulted in the impairment of eosinophils recruitment to the cornea. Therefore, they concluded that ICAM-1 has a selective role in eosinophil transmigration to the corneal stroma on the limbal vessels [46].

### 2.5. Dirofilaria immitis

As for *D. immitis*, Mf, as well as the adult stage of the parasite (that lives in canids’ pulmonary arteries), are in constant contact with the host endothelium. The expression of ICAM-1 and CD31/PECAM-1, but not VCAM-1, was increased in human endothelial cells (HAAE-1) when treated with *D. immitis* adult somatic antigens [47]. 

Furthermore, Morchón et al. [47] compared the stimulatory capacity of adult somatic antigens of *D. immitis* (DiSA) and the recombinant form of their endosymbiont’s protein—*Wolbachia* surface protein (rWSP)—in in vitro cultures of vascular EC. Their results indicate a different stimulatory activity of the two antigens. Both DiSA and rWSP stimulate the production of the enzymes responsible for arachidonic acid metabolism, cyclooxygenase-2, 5-lipoxygenase (5-LO), and leukotriene B4, but only DiSA stimulated the production of prostaglandin E2. As for the adhesion molecules, DiSA stimulated the expression of ICAM-1 and CD31/PECAM-1, whereas rWSP stimulated the expression of ICAM-1, CD31/PECAM-1, VCAM-1, and E-cadherin. Neither of the two antigens (DiSA nor rWSP) altered the basic physiological mechanisms of EC, such as cell proliferation, cell cycling, or apoptosis [48].

## 3. VEGF/Cell Proliferation

Vascular and lymphatic EC themselves can modulate the immune response by producing pro-inflammatory cytokines and chemokines, in addition to several angiogenic mediators, as reviewed by Shao et al. and Card et al. [49,50]. Vascular endothelial growth factors (VEGFs) [51] are signaling proteins upregulated during neovascularization and key mediators of angiogenesis, promoting increased vessel permeability and the inhibition of the host immune response [52].

Recent studies on the molecular mechanisms controlling the lymphatic vessels have shown that vascular endothelial growth factors (VEGF) specifically control lymphangiogenesis in humans by activating VEGF receptor-3, which is principally restricted to the lymphatic endothelium in adults. In animal models, the overexpression of VEGF-C in the skin of transgenic mice resulted in lymphatic endothelial proliferation and the dilation of lymph vessels with a resemblance to lymphatics infected with Mf. Additional evidence for the role of VEGF-C/VEGF-D/VEGFR-3 in the pathogenesis of lymphatic dilation and lymphedema stems from experimental studies in transgenic mice with skin-specific overexpression of soluble VEGFR-3 (sVEGFR-3) using a keratin 14 transgenic promoter. In this genetic model, sVEGFR-3 is secreted at high levels by basal epidermal keratinocytes and binds the lymphangiogenesis factors VEGF-C and VEGF-D, thereby preventing them from activating membrane-bound VEGFR-3 on lymphatic endothelium [53].

### 3.1. Brugia malayi

Live, intact Mf do not stimulate the expression of the angiogenic mediator VEGF-A in vascular EC, but they do stimulate an increase of pro-angiogenic COX-2 human lung microvascular endothelial cells [36]. Bennuru et al. showed that lymphatic EC proliferate in response to adult, but not microfilarial, antigens, and live parasites can induce tube formation by LEC in a contact-dependent manner. *B. malayi* microfilarial antigens also induced a number of angiogenic mediators in LEC [54]. Interestingly, Jeeva et al. detected a *B. malayi* Asparaginyl tRNA synthetase having properties identical to VEGF, leading to vascular EC proliferation, vasodilation, and angiogenesis [55]. 

In contrast, Rao et al. reported an in vitro treatment with fresh excretory–secretory products of adult *B. malayi*, particularly females, significantly suppressed the vascular endothelial proliferation of HUVEC [56]. They observed that the soluble extract of adult worms also decreased HUVEC proliferation, but less significantly than live adult excretory–secretory products. Interestingly, HUVEC treated with lymph from dilated lymphatics of mice infected with *B. malayi* demonstrated increased proliferation [56].

Tetracyclines were reported to have a strong anti-parasitic effect against filarial nematodes. Firstly, by acting against *Wolbachia*, an obligatory endosymbiont of filaroids. Secondly, Furlong-Silva et al. demonstrated by using a murine hind-limb model of filarial infection that doxycycline has anti-lymphangiogenic proprieties by blocking lymphatic EC proliferation in response to VEGF stimuli, which significantly reduces the magnitude of lymphatic remodeling and dysfunction induced by *B. malayi* filarial infection [9].

### 3.2. Wuchereria bancrofti

Although an increase in the expression of angiogenesis and lymphangiogenesis mediators in the sera of humans infected with *W. bancrofti* suggests that lymphatic filarial parasites may directly influence inflammation and angiogenesis, Mf antigens did not cause any significant increase or decrease in expression of VEGF-A, VEGF-C, VEGF-D, and VEGF-R measured by qPCR in vitro [54]. This may be due to the fact that Mf antigens, not live Mf, were used, since, in Polynesian patients living in regions endemic for *W. bancrofti* chyluria (a condition in which chyle is present in urine; mainly caused by Mf leading to the rupture of dilated abdominal lymphatics into the urinary excretory system), was associated with increased VEGF levels, whereas elephantiasis was characterized by an increase in endothelin-1 (ET-1) [45]. Another study showed that VEGF-A plays an important role in hydrocele in men [57], while VEGF-C and VEGF-D play a crucial role in lymphangiogenesis in both men and women [58,59] by activating the VEGF receptor-3 (VEGFR-3) [53,60]. It has been reported that a doxycycline-based treatment targeting *Wolbachia* in *W. bancrofti* decreased the plasma levels of VEGF-C/sVEGFR-3/VEGF-C, which improved the pathology in hydrocele patients [53,61]. On the other hand, Bennuru et al. reported that elevated plasma levels of VEGF-A, VEGF-C, VEGF-D, and angiopoietins (Ang-1/Ang-2) in both microfilaremic and in patients with lymphedema are associated with *W. bancrofti* infection, but not with Wolbachia itself, as they did not change after doxycycline treatment [62]. Finally, Panda et al. reported that a genetic polymorphism in endothelin-1 (a major angiogenic factor) was associated with elephantiasis or hydrocele in patients having clinical manifestations of the infection [63].

### 3.3. Onchocerca volvulus (River blindness)

There are several VEGF receptors (VEGFRs) expressed by the cornea that serve as “decoy” receptors for proangiogenic VEGF molecules. The corneal epithelium displays membrane-bound VEGFR-3 that binds and sequesters VEGF-C and -D. This directly suppresses lymphangiogenesis by inhibiting VEGF-C and -D-mediated signaling, and indirectly suppresses hemangiogenesis by inhibiting the recruitment of VEGF-secreting macrophages. Soluble VEGFR-1 and VEGFR-2 are essential for maintaining corneal avascularity under normal homeostatic conditions [64]. Unlike other VEGFs, VEGF-C cannot be upregulated by hypoxia, but only by proinflammatory cytokines [65]. Briefly, immune cells produce VEGF-C, which stimulates the proliferation of EC acting by VEGF-RC. This process is an important part of lymphangiogenesis, which in turn is significantly involved in the pathogenesis of blindness in onchocerciasis. Cao et al. observed that the injection of recombinant VEGF-C into limbal vessels stimulated neovascularization in mouse cornea in vivo [60]. The levels of the VEGF family and their receptors, Ang-1 and Ang-2, were significantly elevated in patients showing *Onchocerca* nodules in humans in vivo. Other scientists discovered that VEGFR-3 is expressed on corneal dendritic cells (DC) and is likely involved in leukocyte trafficking in the eye, which leads to the loss of vision in *O. volvulus* infections [66].

### 3.4. Dirofilaria immitis

Population-based studies demonstrated an inverse correlation between the number of circulating Mf and the proliferation [67,68]. Zueva et al. [69] created an in vitro model of heartworm disease using dog microvascular endothelial cells to test the effects of *D. immitis* adult antigens on EC. They observed an increase in the expression of proangiogenic factor VEGF-A and that recombinant WSP increased the expression of VEGF [48,70]. Zueva et al. stimulated EC with antigenic extracts from *D.* *immitis* adult worms obtained from dogs infected with *D. immitis*, from which one was treated with doxycycline in order to deplete *Wolbachia* before the experiment. Adult worms from the treated dog contained significantly lower amounts of *Wolbachia* (less than 60%, tested by qPCR) compared to the worms removed from the untreated dog. Only worms from the untreated dog significantly increased the expression of the proangiogenic factor VEGF-A in the EC cultures. However, only worms from the doxycycline-treated dog significantly decreased the expression of the pro-angiogenic mEndoglin and increased the expression of the anti-angiogenic sEndoglin. The higher anti-angiogenic effects mounts of *Wolbachia* in the worms’ antigens induced a proangiogenic response. Therefore, the authors concluded that *D. immitis* worms can stimulate angiogenesis and identified their endosymbiont bacteria, Wolbachia, as a key element in this process [69]. On the other hand, Zueva et al. reported one year later that recombinant surface protein of *Wolbachia* alone reduced the expression of mEndoglin (pro-angiogenic), increased that of sEndoglin (anti-angiogenic), and decreased the trend to the formation of pseudo-capillaries in dog microvascular ECs, which would suggest that this particular *Wolbachia* protein has an anti-angiogenic effect [70]. 

### 3.5. Dirofilaria repens

Ilyasov et al. detected a clear remodeling of the blood vessels, using Doppler, in the periphery of *D. repens* nodules in patients presented with subcutaneous dirofilariasis [71]. 

## 4. Plasmin

Tissue plasminogen activator (tPA) is an enzyme produced by, among others, EC that catalyzes the conversion of plasminogen to plasmin; thus, it is the major enzyme responsible for clot breakdown [72]. Plasmin can activate the lymphangiogenic growth factors VEGF-C and VEGF-D, and modulate the effects of VEGF [73]. Its upregulation is associated with inflammation, as it has been observed that tPA-deficient mice were resistant to post-trauma neuronal degeneration [74,75]. Additionally, plasmin was found to be involved in the M1 to M2 switch of macrophages [76,77].

The ability of plasmin to degrade fibrin, ECM, and connective tissues [78] facilitates the migration and invasion of parasites [79]. Furthermore, plasmin promotes the activation of the complement system, but degrades complement component 5. This, on the other hand, prevents complement component 5b deposition and membrane attack complex formation [79]. Furthermore, plasmin cleaves complement component 3b to an inactive form, which prevents the formation of the terminal complement complex. Unfortunately, to date, there are no reports of whether Mf or adult filarial parasites employ this mechanism. However, it is potentially likely as Mf were found to be coated with C3b and/or iC3b, as described in Section 2.1. Finally, it can be speculated that the recruitment of plasminogen on the worm’s surface is a strategy enabling invasion and survival within the host.

### 4.1. Dirofilaria immitis

*D. immitis* ES upregulate tPA expression in HAAE-1 vascular endothelial cells [80,81]. These molecules, as well as surface-associated molecules and endosymbiont WSP protein, were shown to bind plasminogen and enhance its activation into plasmin [82,83]. Further, González-Miguel et al. [80,81] observed that DiES induce an increase in the expression of tPA in EC in vitro. The overproduction of plasmin (counteracting clot formation) promoted microvilli proliferation in the vessels in cardiopulmonary dirofilariasis. It is believed to be used as the parasites’ survival mechanism and is likely beneficial for the host since it prevents clot formation [79].

### 4.2. Brugia malayi

In 1991, Foster et al. hypothesized that *B. malayi* Mf antigens release factors that inhibit the activation of hemostatic mechanisms [84]. In detail, they observed that Mf antigens completely inhibited the activation of factor XII and platelet aggregation.

## 5. Cytokine Production

Cytokines are known to play an important role in trans-endothelial migration. Nonetheless, there is a limited amount of research that investigated the endothelial cell–filaria cross-talk and, as such, in this review we will discuss the available data. For example, IL-10 suppresses the release of pro-inflammatory cytokines by immune cells [85]. Furthermore, it inhibits the growth of EC and endothelium-dependent T cell stimulation [86]. Interestingly, IL-10 was shown to be produced by human mononuclear cells and T cells challenged with *W. bancrofti* or *Dirofilaria* spp. antigens [87]. In humans infected with *Wuchereria* spp. or *Dirofilaria* spp., lymphocyte proliferation and activity against these parasites’ antigens obtained from male and female adult worms were seen to be significantly lower [67]. 

TGF-B is produced by alternatively activated macrophages and T regulatory cells. Additionally, T cell clones isolated from peripheral blood of individuals with generalized onchocerciasis have a T regulatory cell phenotype [88,89]. As such, they selectively produce IL-10 and TGF-β, but not IL-2 [88,89]. Similarly, T regulatory cells were predominant in experimental models of filarial infection, as CD4+ cells showed elevated expression of CD25+, CTLA4, and glucocorticoid-induced TNFR-related protein (GIFR) [90]. Furthermore, in vivo injection of anti-CTLA4 and GIFR restored cytokine production and reduced parasite survival, consistent with the role of these cells in maintaining a state of immunotolerance [90]. 

Schroeder et al. [36] performed a wide screening of cytokines and chemokines produced by HUVEC in the presence of *B. malayi* Mf using culture supernatants for protein expression array. However, they did not observe Mf presence to have significantly altered the secretion of any molecules tested. Furthermore, the presence of live Mf did not seem to affect the proliferation of extravasated lymphocytes, T cells, CD8+, nor NK cells. However, Mf significantly inhibited the trans-endothelial migration of co-cultured neutrophils and monocytes [36].

To determine whether *Wolbachia* endosymbionts were responsible for any angiogenic mediator induction in EC, Schroeder et al. [38] cultured intact Mf or *Wolbachia-*depleted Mf with HUVEC. The results revealed that angiogenic factors were not altered in the presence of either intact Mf or *Wolbachia*-depleted Mf. Further investigations showed that, in the presence of Mf, neutrophils were more strongly attracted to HUVEC than lymphocytes [36].

In contrast to the effects provoked by the extracts of *B. malayi* Mf [91,92], Schroeder et al. [36] reported that live *B. malayi* Mf can initiate immune responses in their local vascular environment, and thus do not induce a significant increase of pro-inflammatory immune mediators from EC, such as IL-6, TNF-α, or IL-1b. Schroeder et al. also observed that live Mf did not induce increased levels of IL-13, known for promoting alternatively activated macrophages (AAMø), or the down-regulatory cytokines IL-10 or TGF-b1. However, Mf presence did induce the mRNA expression of the inflammatory complement component, C5, in HUVEC, but not human lung microvascular endothelial cells. On the other hand, both monocytes and neutrophils can kill Mf via the production of reactive intermediates. However, Mf can partially neutralize the toxic effects of these intermediates by secreting anti-oxidant enzymes, such as peroxidases and superoxide dismutase [36,92,93,94,95].

IL-8 and CCL2 are considered to be key chemokines for the trans-endothelial migration of neutrophils and monocytes [96]. However, Mf presence seems to have no effect on IL-8 or CCL2 production by EC [36]. This was confirmed by Weinkopff et al., who observed that filarial excretory–secretory products alone were not able to activate the LEC [97]. Further experiments performed by Weinkopff et al. showed that ES indirectly activate the LEC by inducing human peripheral blood mononuclear cells, specifically monocytes, to produce lymphangiogenic factors, such as IL-6, IL-8, and VEGF-A, which induce the formation of LVs in vivo [97].

Morchón et al. [98] also reported that the bacterial obligatory endosymbiont *Wolbachia* (detected in almost all species of filarial nematodes) plays an important role in the inflammatory pathology of filariasis. They reported an intense expression of mRNA of inducible nitric oxide synthase (iNOS) and increased production of intravascular NO (inflammatory mediator of the innate response), as well as IFN-γ and a Th1-profile of antibody response in BALB/c mice immunized with the *Wolbachia* surface protein [98].

Finally, the parasite lives in the lymphatics of the mammalian host in close proximity to lymphatic endothelial cells, which are known to express iNOS and to generate prodigious quantities of NO in response to cytokines. The adult-stage antigen-specific immune response of immunocompetent mice can generate the requisite cytokines in response to specific filarial antigens and induce NO release in proximity to the parasite. This would explain the failure to resist *B. malayi* infection by wild-type mice treated with an inhibitor of NO synthase compared to complete resistance to infection in immunodeficient mice treated with a compound that releases NO demonstrated by Rajan et al. [99].

## 6. Conclusions

The intriguing interaction between filarial nematodes and the host vascular and lymphatic endothelium is a hallmark of filariasis in humans and animals. The endothelium–parasite crosstalk likely promotes the parasite survival and its potential transmigration through the blood wall vessel to the tissues’ capillaries by increased expression of adhesion molecules, VEGF, and plasmin production. It seems that the parasites are able to inhibit the trans-endothelial migration of neutrophils and monocytes and promote the release of immunosuppressive cytokines, such as IL-10, which inhibit T cell activation.

In this review we discussed the effects of live parasites or their antigens on the host cells. Undeniably, live parasites allow a more precise and truthful observation of the parasites’ pathological effect on the endothelium. However, the antigens of the adult parasites may allow an assessment of the process occurring while parasites die, and the use of a selected recombinant protein permits to study their proprieties as biotherapeutic agents. In order to evaluate host–parasite interactions, the use of live parasites is obviously the most relevant model, but not for all discussed parasites these data existed. Additionally, some studies separated and compared the effects of filarial nematodes containing their obligatory *Wolbachia* endosymbiont and *Wolbachia-*depleted worms on EC. These studies revealed that *Wolbachia* is likely involved in the promotion of angiogenesis [69]. 

The divergence of findings obtained by several groups is likely associated with the use of (1) excretory–secretory products from adult worms vs. Mf or (2) live adult worms vs. Mf (3) different cell lines from various species (human/murine/canine). In order to understand the true parasite–EC cross-talk, the use of live parasites of Mf embryo larval and adult stages challenging primary endothelial cells would allow for the most accurate assessment of the effects of filarial nematodes on EC.

## Data Availability

Not applicable.
